# Healthcare in Equatorial Guinea, West Africa: obstacles and barriers to care

**DOI:** 10.11604/pamj.2014.19.369.5552

**Published:** 2014-12-10

**Authors:** Kim Eleanor Reuter, Aurora Geysimonyan, Gabriela Molina, Peter Robert Reuter

**Affiliations:** 1 Temple University, Department of Biology, Philadelphia, PA, 19122, USA; 2 California State University Fullerton, Department of Public Health, Fullerton, CA, 92831, USA; 3 Florida Gulf Coast University, College of Health Professions & Social Work, Fort Myers, FL, 33965, USA

**Keywords:** Equatorial Guinea, africa, healthcare worker, perception, attitudes

## Abstract

**Introduction:**

The provision of healthcare services in developing countries has received increasing attention, but inequalities persist. One nation with potential inequalities in healthcare services is Equatorial Guinea (Central-West Africa). Mitigating these inequalities is difficult, as the Equatoguinean healthcare system remains relatively understudied.

**Methods:**

In this study, we interviewed members of the healthcare community in order to: 1) learn which diseases are most common and the most common cause of death from the perspective of healthcare workers; and 2) gain an understanding of the healthcare community in Equatorial Guinea by describing how: a) healthcare workers gain their professional knowledge; b) summarizing ongoing healthcare programs aimed at the general public; c) discussing conflicts within the healthcare community and between the public and healthcare providers; d) and addressing opportunities to improve healthcare delivery.

**Results:**

We found that some causes of death, such as serious injuries, may not be currently treatable in country, potentially due to a lack of resources and trauma care facilities. In addition, training and informational programs for both healthcare workers and the general public may not be effectively transmitting information to the intended recipients. This presents hurdles to the healthcare community, both in terms of having professional competence in healthcare delivery and in having a community that is receptive to medical care.

**Conclusion:**

Our data also highlight government-facility communication as an opportunity for improvement. Our research is an important first step in understanding the context of healthcare delivery in Equatorial Guinea, a country that is relatively data poor.

## Introduction

The provision of healthcare in developing countries has received increasing attention, but still presents challenges to the international community. For example, a 2009 report noted that 99% of female mortalities during pregnancy and childbirth were in developing nations, that only 30% of HIV positive individuals received treatment in developing countries, and that the availability of medicines in these areas was poor [1]. Addressing these healthcare disparities has been a key objective of initiatives like the Millennium Development Goals [1]. While these initiatives have resulted in the lowering of some disease incidence rates [1],health sector inequalities are still present within [2, 3] and among developing countries [4]. Data can increase understanding and help address healthcare inequalities, and data collection is key to equalizing healthcare systems in these areas. For example, past studies have found that the manner in which inequalities in healthcare manifest in developing countries is not intuitive, but that data can clarify these misconceptions and allow development decisions to be made with more certainty [4, 5]. However, a paucity of data still exists across many developing nations [1, 5].

Qualitative data - which can include understanding healthcare systems from the perspectives of caregivers, healthcare workers, and patients - help capture the context in which a healthcare community functions. Qualitative studies provide interesting insights about the real and perceived hurdles that healthcare professionals face in their work environments, and how these change across cultures. For example, qualitative studies have found that tardiness [6], mortality rates, medical errors, and a patient's length of stay [7] may increase when healthcare professionals hold a negative opinion of their professional environments. Other studies have examined South African nurse practitioners’ perceptions of traditional medicine [8], job satisfaction in Senegal and Mali [9], Ugandan healthcare worker quality of life [10], and the influence of healthcare provider opinion's on the likelihood of prescribing antibiotics in Lesotho [11]. Even a nurse's nationality can impact his/her self-reported levels of caring for patients and views on the use of technology in medicine [12]. These examples highlight how qualitative data can substantially increase understanding of why regional and national healthcare systems face certain problems and provide a context for interpreting disease prevalence, public health statistics, and mortality trends. Equatorial Guinea (Central-West Africa) is one country whose healthcare system and public health needs remain relatively underexplored, despite the collapse of its medical system in the mid-1970s [13]. The reasons for this lack of data are complex but may be linked to the country's closed borders until the late-1970's [13], the ongoing difficulties of undertaking field work and research projects in-country (KER, personal observation), and the lack of reliable baseline data including common measures of public health metrics [1].

Past studies in Equatorial Guinea have focused on issues often linked to large-scale, development initiatives. For example, there is a relative abundance of papers discussing malaria transmission and prevention [14], of medical case studies observed in Equatoguinean facilities [15], and reports on government-funded monitoring programs [16]. These are supplemented by prevalence and morbidity data reported in a few published papers [17, 18] and summarized by the World Health Organization [1, 19]. These are informative but occasionally incomplete [1], outdated [16, 17], or presented with a degree of uncertainty [19]. Missing are attempts to understand the system from the perspective of healthcare workers, which could provide valuable insight into the practical reality of a country's healthcare strategy. To our knowledge, there are no qualitative studies examining the Equatoguinean healthcare system or potential obstacles and barriers to care. The lack of these types of studies are concerning, as 76.8% of the population lived in poverty in 2006 [20], primary healthcare providers are available sporadically only across the country [21], and social assistance programs for at-need communities are not well established [22]. We aimed to fill this knowledge gap by interviewing members of the Equatoguinean healthcare community. Our two objectives were to: 1) learn about the most common diseases and the most common causes of death in Equatoguinean communities from the perspective of healthcare workers; and 2) gain an understanding of the healthcare community by describing how, a) healthcare workers gained their professional knowledge, b) summarizing ongoing healthcare programs aimed at the general public, c) discussing conflicts within the healthcare community and between the public and healthcare providers, d) and addressing opportunities to improve the system.

## Methods

**Figure 1:**
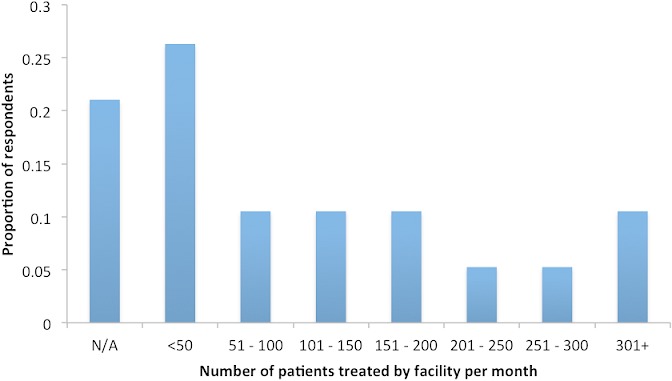
Number of patients treated per month in facilities in which respondents worked

*Respondents*: For objective one, 18 respondents were surveyed using semi-structured interviews [23] in March-April (n = 12) and June 2013 (n = 6). Respondents had held their positions for 7.9 ± 6.6 years (range: 1-20 years) and represented facilities with a wide range of patient loads (Figure 1). Respondents interviewed in June were part of an extended study that aimed to describe the country's mental health system, and were directors or administrators in hospitals and at top levels of government. For objective two, respondents interviewed in March-April were spoken with for 36 ± 11 minutes (mean ± st. dev, range: 10 - 53). These 12 respondents held the following position(s): nurse aid (n = 1), nurse (n = 3), health worker (n = 2), doctor (n = 2), or director/chief of a health center (n = 4). Most (n = 10) respondents treated patients of all ages. Seventeen interviews were conducted in Spanish and one interviewee chose to be interviewed in English. Interviews were conducted in private by one Spanish-speaking interviewer (AG), at a time and place determined by the interviewee. The interviewer was not known to the interviewee and was not local to the area.

*Survey Instrument*: We asked open-ended questions (always preceded by the phrase “from your perspective”) including: 1) What are the most common diseases treated in your facility?; 2) What are the most deadly diseases treated in your facility?; 3) What types of healthcare knowledge are most useful to you in your current position? Where did you initially obtain this knowledge?; 4) Are there any ongoing healthcare activities in the area?; 5) Are there actions taken within the community or by any ongoing healthcare activities which conflict with each other?; 6) What are the main obstacles to successful healthcare activities in this area? What are the barriers that you face, in your healthcare activities?; 7) How would you improve existing healthcare activities?; 8) What are the major opportunities for healthcare advancement in the area? To ensure interviewee comfort, not all respondents were asked all questions and not all respondents answered all questions. Therefore, sample sizes vary, but are clearly indicated in the results.

*Ethical considerations*: Though identifying information was collected and interviews were recorded, respondents are not identified to protect anonymity. Written consent was secured. Research was approved by an ethical research board (Institutional Review Board at Florida Gulf Coast University) and by the teaching and research faculty at the University of Equatorial Guinea Medical School. All interviews were conducted by a researcher trained in ethical data collection and followed all national laws relevant to the survey of adult populations.

*Statistical Analysis*: Given the qualitative nature of the interview data, and given the small sample size, data were coded to illustrate trends but were not analyzed quantitatively.

## Results

### Common & deadly diseases/afflictions

**Figure 2:**
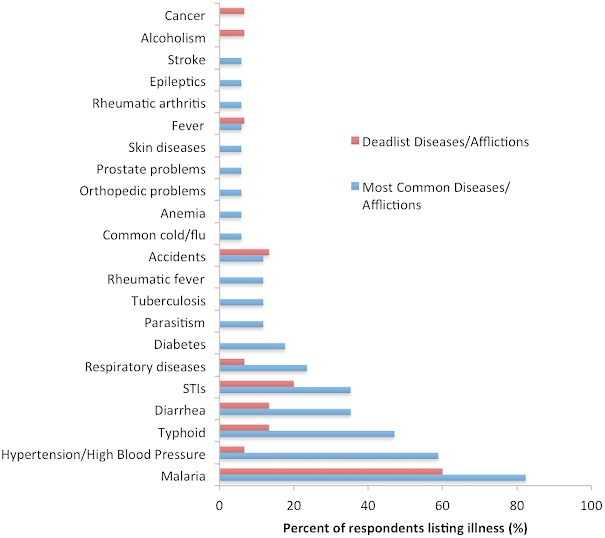
Common and deadly diseases and afflictions in Equatoguinean communities, as reported by respondents. Accidents included automobile accidents in urban areas and agricultural accidents in rural areas. Of the six people who mentioned sexually transmitted infections (STIs), HIV/AIDS was mentioned most frequently (n = 5), followed by hepatitis (n = 2), syphilis (n = 2), gonorrhea (n = 1), and chlamydia (n = 1)

Sixteen respondents mentioned 20 diseases/afflictions, when asked to list the most common diseases in their communities (Figure 2). Fourteen of fifteen respondents who were asked, listed the most deadly diseases facing their target communities (Figure 2). Fever, malaria, and diarrhea were mentioned by at least one respondent each for being especially deadly in children.

### Healthcare in Equatorial Guinea

*Healthcare knowledge*: 13 respondents were asked to describe the types of health knowledge most useful to them. Responses included: basic knowledge of symptoms (n = 6), understanding the link between environment and health (n = 2), prenatal and pediatric knowledge (n = 2), and sex education (n = 1). The emphasis by 46% of the respondents on the importance of knowing basic symptoms was explained by one individual who noted the lack of other diagnostic tools and laboratories. The link between the environment and health was mentioned in the context of malaria transmission, community sanitation, and contamination of community water resources by human waste; landfills, protection of water sources, and ensuring clean latrines were also mentioned by several respondents in response to this and other questions. One respondent indicated that a large proportion of people were not well informed about sexually transmitted infections (STIs).

*How did you initially obtain your health knowledge?* Five of the thirteen respondents asked, provided the following responses: from colleagues (n = 5); school, university, or a professional course (n = 3); government-sponsored preparation course (n = 1); and lectures (n = 1).

*What are the ongoing healthcare activities in the area?* Twelve respondents were asked and provided the following list of in-country healthcare activities: seminars/lectures (n = 8); anti-malaria campaigns (n = 4); vaccination campaigns (n = 2); epidemics response (n = 2); breast-feeding outreach (n = 1); tuberculosis monitoring (n = 1); diabetes program focusing on control and prevention (n = 1); call-in radio program where questions are answered by healthcare professionals (n = 1); and a mobile clinic (n = 1).

Though healthcare seminars and lectures were frequently mentioned, respondents were not clear if these were successful in their aims of conveying information to the public. Lecture topics included: malaria (n = 3); family health, family planning, and pregnancy (n = 3); HIV/AIDS (n = 2); diarrhea (n = 1); and sanitation topics including hand washing, food preparation, and food expiration dates (n = 1).

Outreach campaigns were also described. The vaccination campaigns (n = 2 respondents) focused on childhood vaccination and the provision of tetanus shots to pregnant women. Campaigns used radio and television transmission, were described as ongoing, successful, and long-term with an impact of several thousand people, using mobile clinics to reach rural populations. Vaccinations were described as an accepted form of modern medicine. A campaign that was not considered successful (n = 1 respondent) involved World Health Organization efforts to encourage breastfeeding in the first six months after birth. The respondent reported having difficulties explaining the importance of breastfeeding and noted that mothers wanted to bottle-feed children.

Some respondents described community-monitoring programs. Examples included: 1) a tuberculosis monitoring program of communities for symptomatic residents who were directed to hospitals for testing (n = 1 respondent); 2) epidemics response from the Ministry of Health to train healthcare workers in identifying symptomatic community members (n = 2); and 3) free treatment for communities during “diarrhea epidemics” that successfully saved lives (n = 1).

*Healthcare conflicts within the community*: Twelve respondents were asked about conflicts within the community. Seven respondents (58%) reported conflict(s): between traditional and modern medicine (n = 2); non-compliant patients (n = 2); communication difficulties with patients (n = 1); cultural issues such as polygamy increasing STI transmission (n = 1); unhealthy lifestyles habits (n = 1); alcoholism (n = 1); issues with government bureaucracy (n = 1).

The conflict between traditional and modern medicine was raised several times in response to these and other questions, usually as an example of a negative healthcare outcome. However, one respondent explained that healers and western medicine could work together, usually on topics (e.g. mental health) that are difficult to treat in hospitals and for which the community usually prefers traditional healers.

A method to increase patient compliance was suggested by one respondent who felt that patients might follow medical advice if large-scale outreach campaigns were instituted by the government using mass media; these could explain how medical advice and preventative measures can result in decreased incidents rates of disease and preventable illnesses. A second respondent felt that out reach could teach people, “that certain diseases need good nutrition and good habits.” The concept of disease prevention was raised by a third respondent who noted that, “prevention is a new concept in this country.”

Finally, communication difficulties between patients, communities, and medical practitioners were raised as an issue (n = 1 respondent); the community's use of various languages and lack of comprehension of basic medical terms decreased effective communication between healthcare workers and patients.

*Obstacles and barriers to care*: Ten respondents were asked about obstacles and barriers to successful healthcare activities and their professional responsibilities. Nine respondents listed obstacles to care, such as: non-compliance with hygiene regulations (n = 4); infrastructural concerns (n = 1); alcoholism (n = 1); and a lack of awareness in the community (n =1); resources (n = 3); pay (n = 1); and healthcare coverage across all regions of the country (n = 1).

Respondents seemed frustrated by communities’ that did not follow hygiene guidelines. One respondent explained that the population treats health compliance as a legal/government issue despite outreach explaining that basic sanitation could increase patient wellbeing. Another respondent echoed these sentiments, saying that communities did not accept preventative care or sanitation guidelines. One respondent simply summarized that there was, “a lack of a culture of prevention.”

Several types of resource shortages exist. One respondent indicated that the clinic was too small for its regular patient load, another indicated that more trained staff were needed, while a third respondent specified the need for more laboratory supplies, including reagents required for diagnostic tests. One respondent observed that no typhoid or hepatitis vaccines are available (citing WHO regulations as the reason); another noted that condoms are often in short supply.

Lack of pay was a deterrent to one respondent who had not been paid for six years, causing colleagues to leave the profession. Lack of funding was also cited as the reason why another respondent described difficulties in being able to provide care; patients’ payments could not keep facilities fully funded and government assistance to cover administrative costs was insufficient.

*How would you improve existing healthcare activities?* Six respondents were asked and responded with suggestions for how healthcare activities could be improved: more medicines and resources (n = 6), better government-level planning and communication (n = 4), training opportunities (n = 1), and raising community awareness (n = 1).

All respondents indicated a need for more free medicines and resources. One respondent noted that despite treatment being free, the cost of medications decreased a patient's willingness to receive timely care. Cost as a deterrent to care was echoed by a second respondent for rural patients who are sent to urban centers for testing-due to resource shortages in rural hospitals-who may not pursue follow-up care due to the costs incurred. A third respondent stated that transportation is sometimes not available even when rural patients are willing to seek treatment. Several respondents observed a general lack of trained doctors.

Eighty percent of respondents indicated that better government-level planning would help with the delivery of care. One respondent said that an irregular delivery of government-sponsored resources and medicines was a barrier in HIV/AIDS testing and care. Another respondent said that, “it would be nice if the Ministry sends an action plan and gives us a certain amount of money (that) could cover our costs.” A third respondent noted that it may often appear to centralized government agencies that hospital staff do not want to work, when they actually do not have the tools to complete tasks. Several respondents (n = 4) mentioned having requested resources from government agencies and either not receiving them, receiving expired medications, or not getting a response to those requests.

Finally, one respondent asked for more training opportunities, noting that the irregularity of training and provision of supplies often resulted in staff forgetting how to complete laboratory tests and a general decrease in laboratory competence.

## Discussion

### Common & deadly diseases/afflictions

Our data highlight what diseases communities are receiving treatment for, and what diseases -from the perspective of healthcare workers - are most common and the most common causes of death; some of the diseases mentioned by respondents are not regularly included in quantitative datasets. Our respondents’ observations matched the incidence rates reported by the World Health Organization; for example, malaria was cited as a common disease more frequently than HIV/AIDS or tuberculosis. Currently, the malaria incidence rate is 24,767 per 100,000 people, as opposed to 4,259 infections per 100,000 people for HIV/AIDS, and 164 infections per 100,000 people for tuberculosis [24].

However, the diseases that respondents viewed as deadly did differ from what might be inferred from statistics. The deadliest disease listed by most of our respondents was malaria, which causes relatively few deaths per year (220) [1] among the thousands who are infected [24]. For comparison, tuberculosis - which caused 87.5 deaths per 100,000 [1] out of the 164 infections per 100,000 people [24] - was not listed by respondents as a deadly disease. In addition, the listing of typhoid as a deadly disease is interesting as it can be prevented by vaccination, though one respondent's observation about the difficulties of importing typhoid vaccinations may explain why this is the case. Finally, death due to injury - estimated to cause 10% of all Equatoguinean deaths [19] - may highlight the lack of trauma and emergency care in the country. Respondents were especially concerned about automobile accidents in urban areas, agricultural accidents in rural regions, and estimated that the majority of incidents were alcohol-related.

### Training of healthcare professionals & community outreach

Although respondents received academic and professional training, they did not mention if continuing education opportunities are routinely available. While few respondents mentioned training opportunities as a method for improving the healthcare system, continuing education programs could reiterate basic health knowledge and common disease symptoms; topics deemed important by approximately half of the respondents.

In contrast, an extensive number of public healthcare outreach programs have been provided to the community. It is clear that a wide range of programs are being organized for the general public and that the topics are typically related to common or deadly diseases, though respondents routinely thought that more outreach was needed. Notably, no respondents listed outreach programs related to hypertension prevention (listed as both a common and a deadly disease by respondents) or for alcoholism, which was cited as a deadly disease, a barrier to healthcare, and linked to automobile accidents. This could indicate that these two issues are not the focus of public outreach programs, despite the burden that they may place on the healthcare system, as 36% of Equatoguinean adults have elevated blood pressure [19]. Another recurring theme was the need for communities to understand the role that prevention plays in their personal care, and also the link between hygiene and health.

For outreach to be effective, it should increase not just the number of programs, but the quality as well. There is evidence to suggest that current outreach programs are not effectively communicating information to the public. One respondent described a breast-feeding campaign that may not have properly prepared healthcare workers to discuss breastfeeding in the context of the local culture. The consequences of this anecdote are highlighted by data showing that the duration of breastfeeding has been decreasing in Equatorial Guinea despite WHO recommendations [18]. In addition, it is not clear that steps have been taken to mitigate conflicts between modern and traditional medicine in a systematic way, despite the fact that this conflict in Equatorial Guinea has been noted before [25]. Finally, public lectures focused on issues related to sanitation and HIV/AIDS may not have targeted communities effectively as communities are reportedly not accepting hygiene regulations, and respondents felt that patients often did not know basic sexual health facts. These anecdotal observations are confirmed by data indicating that only 4% of Equatoguinean women aged 15-24 had a comprehensive, correct knowledge of HIV/AIDS; markedly lower than the regional average of 23% [1].

### Barriers to care and opportunities for improvement

Over half of respondents reported conflicts between the community and healthcare facilities and 90% could name obstacles and barriers to the provision of care. The resource and staff shortages, as described by respondents, are endemic to many sub-Saharan countries [26]; Equatorial Guinea only has three doctors per 10,000 people [1]. Likewise, concerns regarding sanitation and community hygiene are supported by data that many Equatoguineans (43% and 51%) do not have access to improved drinking water and improved sanitation [1]. The utilization of one water source for multiple purposes has also been noted in prior research [16]. However, respondents provided insights into how the Equatoguinean system could be improved. Specifically, respondents highlighted streamlining government oversight and interaction with healthcare facilities as an opportunity for a relatively low-cost investment into the healthcare system. It should be noted that current methods of communication between the government and healthcare programs was cited as a conflict in the healthcare community, a barrier to care, and a place where existing healthcare activities could be improved.

Communication between healthcare facilities and the government could be improved in several ways. This would be a relatively inexpensive method of minimizing some of the administrative hurdles faced by healthcare facilities. Interventions could focus on: (1) providing action plans detailing dates of resource delivery (e.g. medicines) and amounts of financing to be provided; (2) standardizing request procedures for resources and ensuring automatic response to all requests; (3) and assisting facilities in requesting aid from non-governmental groups and private organizations to supplement resources. Other useful, though more expensive, changes could also be instituted to streamline government-facility interaction, including: (1) regular salary dispersal for healthcare workers, with a clear communication in times of non-payment; and (2) providing resources to rural hospitals to enable patient care, or clearly designating facilities that are or are not able to provide specific services. Other examples of government-funded services that would lower the barriers to care include: (1) providing low-cost/free transportation options to patients, and (2) additional countrywide outreach campaigns.

### Strengths and weaknesses of the study

The difficulties of working in Equatorial Guinea where many professionals are not comfortable speaking - even anonymously - about government-related programs limited the scope of the study. We were able to interview only a small number of individuals and we were advised to keep questions broad and open-ended to decrease interviewee discomfort. These limitations may have biased the results as individuals willing to speak with us may have had a different professional experience than those averse to speaking with researchers. However, given the lack of qualitative data concerning the Equatoguinean healthcare system, we nevertheless believe that the information presented here useful in understanding how the system can be improved.

## Conclusion

Our data show that although available statistics may capture the extent of common diseases, the burden of deadly diseases to the healthcare system may not be adequately quantified. Our data indicate that some causes of death, such as injury, may not be currently treatable in Equatorial Guinea, potentially due to a lack of resources and trauma care facilities. In addition, training and informational programs for healthcare workers and for the general public do exist, but they may not be effectively transmitting information to the intended recipients, either due to the small quantity of programs or to the quality of existing outreach initiatives. These present large hurdles to the healthcare community, both in terms of having professional competence in healthcare delivery and in having a community that is receptive to medical care. Other obstacles to care exist, many of which are found across sub-Saharan Africa, including a lack of resources, staff, and general problems with sanitation. However, our data highlight government-facility communication as an opportunity for Equatoguinean healthcare. If instituted correctly, streamlined communication procedures could alleviate several different, but connected, hurdles faced by healthcare workers. Our research is an important first step in understanding the context of healthcare delivery in Equatorial Guinea, a country that is relatively data poor. However, additional research is needed, particularly in increasing understanding how patients perceive healthcare in their communities, which is an angle that has thus far not been examined.
